# Internet-Based Interventions for Women’s Sexual Dysfunction

**DOI:** 10.1007/s11930-016-0087-9

**Published:** 2016-06-30

**Authors:** Jacques van Lankveld

**Affiliations:** Open Universiteit, Faculty of Psychology and Educational Science, Heerlen, The Netherlands

**Keywords:** Internet-based, Online, Sexual dysfunction, Intervention, Women, Female

## Abstract

The present paper gives an overview of the methodology and results of the first decade of research into Internet-based interventions for women’s sexual dysfunction. The interventions, retrieved in a literature search, were mostly well grounded on common theoretical models of sexual dysfunction and psychological disorders, and most ingredients of the interventions were theory-informed. Most interventions offered Web-based therapeutic content within a more or less preprogrammed structure. Most of these also offered prescheduled and/or participant-initiated contact with a sexual health care professional. Comparative effect studies showed improvements in sexual functioning as well as relational functioning at the point of termination of the intervention period. Improvements at posttreatment were generally maintained for several months after termination of the active intervention period. The results of this review seem to warrant further development of Internet-based interventions for women’s sexual dysfunctions.

## Introduction

The use of the Internet as a service delivery mode in the care for women and men with sexual dysfunctions has a relatively brief history. Internet-based psychotherapeutic interventions for common mental disorders, including anxiety and depressive disorders, have proliferated in recent years [1–3, 4•, 5•]. To a lesser extent, such interventions have also become available in the treatment of sexual dysfunctions. Probably, the first scientific publication was authored by Hall [6] who described the treatment of eight women and men with various sexual difficulties through online task-based psychosexual therapy. The author concluded that the initial outcomes were positive. Since that first publication, several have followed, describing outcome studies of Internet-based interventions for sexual dysfunctions using various research designs. The slow start was notwithstanding the notion that the translation of professionally administered sex therapy to Internet-based delivery modes might have been more easily achieved than for many other common mental health problems [7], given that sex therapy has contained self-help elements ever since its emergence in the 1970s [8], and because the core ingredients of therapeutic change in sex therapy (the sensate focus exercises) were in most cases already performed in the privacy of the couples’ homes [9].

Online help has advantages over face-to-face contact [10]. For some individuals, the anonymity that is sometimes provided during (the initial phase of) online therapy can make it a preferable option compared with face-to-face types of help. The increased accessibility of online help, including the independence of geographic location and, in some modes of delivery, also the asynchronous nature of the contact between client and therapist, may provide a valuable service for those who have difficulties to attend face-to-face meetings due to physical or geographic constraints, or for work-related reasons. Moreover, because treatment is administered in daily-life conditions, generalization of treatment gains to the real-life environment may be less problematic. Finally, improvements in sexual functioning following online interventions might be more attributed to one’s own efforts and competence, and thus boost one’s self-esteem. However, disadvantages and risks are also recognized. One risk factor that Andersson and Titov [10] identified is the currently limited knowledge about who might benefit and who is likely to fail using online help. Treatment outcome predictors that were studied were not very robust. A related topic is that negative outcomes may go undetected, thus leaving sufferers in quandaries.

Reviews and meta-analytic compilations of controlled and randomized outcome studies on thousands of clients have provided abundant evidence for the efficacy and effectiveness of Internet-based care for individuals suffering from anxiety and depressive disorders [11, 12] as well as for health promotion [13••]. Increasingly, evidence is also found for the cost-effectiveness of this approach [11, 14–18]. Thus far, no reviews of the outcome of Internet-based interventions for women’s sexual dysfunctions have been published.

The characteristics of Internet-based interventions can be conceptualized along three main lines [13••]: (a) the theoretical foundation of the intervention and the use of theory-based predictors; (b) the specific strategies and behavior change techniques used in the intervention; and (c) the mode of delivery. In the present paper, the published empirical research of Internet-based therapy for women’s sexual dysfunctions between its introduction and March, 2016 will be reviewed using this conceptual framework.

### Theoretical Background of Internet-Based Interventions

Internet-based interventions can be characterized according to the extent to which they are founded on established theory. Although it is thus far unknown whether the effectiveness of Internet-based interventions is significantly impacted by reliance on established theory [19, 20], it is plausible that theoretical considerations may be beneficial when they are used to guide the selection of disorder aspects that need to be targeted (e.g., learning mechanisms, automatic vs deliberate disorder-relevant cognitions, self-efficacy, self-esteem, partner communication patterns), of techniques to address mechanisms underlying particular problem types (e.g., skills deficits), or the selection of target groups of patients that are most likely to benefit from participation (e.g., with specific clinical diagnoses) [13, 20, 21•]. Clear definition and identification procedures of the target group for which an intervention might be beneficial might prevent incorrect self-diagnosis that could lead one to choose an unhelpful strategy [21•].

### Specific Strategies and Behavior Change Techniques Used in Internet-Based Interventions

Internet-based interventions for sexual dysfunctions employ specific strategies and behavior change techniques to promote therapeutic change. Such strategies and techniques are mostly typical for the underlying theoretical model that guided intervention development (e.g., cognitive restructuring techniques based on cognitive models of sexual dysfunction, see [22–25]) or masturbation training for primary orgasmic problems in women [26].

### Modes of Delivery of Internet-Based Interventions

Internet-based interventions may be fully self-directed and work as a self-help approach, comparable to unassisted bibliotherapy [27, 28], with a structured therapy program encapsulated in a computer algorithm. Others are augmented with varying levels of therapeutic assistance. Furthermore, variations in the use of Internet technology for therapeutic assistance are found: video conferencing utilities are used, as well as text-based technology, both through Internet (e-mail, Whatsapp, snapchat) and telephone, including SMS. Client-therapist contact may be synchronous, such as in video conferencing, or asynchronous, including exchange of both text and video recordings. Internet-based interventions may be implemented as stand-alone interventions or may be blended with other, face-to-face interventions. Additional techniques can involve the use of mobile phone-based data collection (“ecological momentary assessment” [29, 30]) and conditional feedback on mobile monitoring data in both standardized and tailored formats. A coding matrix for assessing mode of delivery in Internet-based interventions developed by Webb and colleagues [13••] will be used to guide the discussion of included interventions.

## Method

In March 2016, a search was conducted using Web of Science, PubMed, and Psychinfo. Search terms in the publication title, combined using Boolean operators, were as follows: (sexual dysfunction* OR sexual disorder* OR sexual difficult* OR sexual problem* OR sexual complaint*) AND (women* OR female OR woman*) AND (Internet OR online OR www OR world wide web) AND (treatment OR therap* OR intervention OR controlled trial OR RCT). This search yielded 16 publications. The reference lists of the retrieved papers were searched for publications meeting the inclusion criteria. One more paper was identified. After reading the abstracts, eight publications were retained for the review. Inclusion criteria were as follows: the study addressed female sexual dysfunctions; the major part of the intervention was delivered via the Internet; participants were allocated using a randomization technique to an active intervention group or to a comparison group receiving a control intervention or no intervention; and a measure of sexual functioning or sexual dysfunction after termination of the intervention was performed. The included publications were searched for details on the treated population, study characteristics, intervention characteristics, assessment instruments used, and treatment effects.

## Results

Eight publications were included [31–36, 37••, 38]. Seven of these reported on four completed RCTs; one paper reported the design of an RCT of which the data collection was still ongoing [34]. Two studies, reported in five publications [31–33, 35, 36], included women from the general population with mixed sexual difficulties, related to sexual desire, arousal, orgasm, or sexual pain and who were living in a stable heterosexual relationship. One study included women with either localized breast or gynecological cancer [37••] and who scored as sexually dysfunctional (under 26.5) on the Female Sexual Function Index [39]. One paper collected qualitative data from gynecologic cancer patients who were randomized to the immediate start of a Web-based support group or a waitlist control group [38]. Other characteristics of the included studies are summarized in Table 1. Characteristics of the study interventions are summarized in Table 2.

**Table 1:** Characteristics of the randomized, controlled outcome studies of Internet-based interventions for women’s sexual dysfunctions

Citation(s) for study; country	Targeted group; *N* = sample size at randomization; (N_INT_) = sample size of active intervention	Intervention type (*Name*); type of control group; duration to intervention assessment	Type of Internet intervention and type and level of human therapist contact	Primary (A) and secondary outcome measures; follow-up duration	Differential effects between active intervention and control group of intervention on outcome measures at *posttreatment* and at *follow-up*: effect size (if effect size not reported: significance level)
Jones and McCabe, [35]; McCabe and Jones, [36]; Australia	Community women: hypoactive sexual desire disorder, sexual arousal disorder, anorgasmia, or genital pain ; *N* = 39 (N_INT_ = 17)	CBT program (*Revive*); delayed treatment; 10 weeks	Regular (weekly) e-mail contact with therapist; additional unlimited client-initiated e-mail contact with therapist	FSFI, DASS, SFS, PAIR; 3 months	Only analyzed in participants who were sexually active at pretest in the past month; *Post-treatment effects*: FSFI: η^2^_part_ = 0.56; PAIR: η^2^_part_ = 0.39; DASS: n.s. *Post-treatment to follow-up effects*: FSFI: n.s.; PAIR: n.s.; DASS: n.s.
Hucker and McCabe, [31-33]; Australia	Community women: Hypoactive sexual desire disorder, sexual arousal disorder, anorgasmia, or genital pain; *N* = 57 (N_INT_ = 26)	Mindfulness-based CBT (*Pursuing Pleasure*); delayed treatment; minimum 11 weeks	Six preprogrammed progressive online modules, with hurdle requirements for transition to next module; therapist-guided, unstructured, real-time text chat group, 1 h every 2 weeks	FSFI, FSDS-R, SFS, PAIR; 3 months	*Post-treatment effects*: PAIR sexual intimacy higher: η^2^_part_ = 0.26; PAIR emotional intimacy higher: η^2^_part_ = 0.08; PAIR communication higher: η^2^_part_ = 0.08; PAIR relational satisfaction: n.s. *Posttreatment to follow-up effects*: PAIR sexual intimacy lower: *p* = 0.02; PAIR emotional intimacy: n.s.; PAIR communication: n.s.; PAIR relational satisfaction: n.s.
Wiljer et al. [38]; Canada	Gynecologic cancer patients reporting sexual distress; *N* = 27 (N_INT_ = 13)	12-week, Web-based support group (*GyneGals*); waitlist; 12 weeks	Asynchronous text-based support group (e.g., bulletin board format or forum) with discussion focusing each week on different, specific topics; forum content monitored daily; one 90-min synchronous, live chat session with a gynecologic oncologist, a radiation oncologist and the forum facilitators	Semi-structured telephone interview (30 min): Participants’ perceptions of feasibility and efficacy of the intervention; 12 open-ended questions on participants’ experiences with website registration and ease of usage, program topics, usefulness of the information provided, helpfulness of forum moderators and support group members, and benefits and satisfaction with the intervention.	Women in the active intervention group reported benefits to participating in the intervention, including receiving support from group members and moderators, increased emotional well-being, improved feelings of body image and sexuality, and comfort in discussing sexuality online.
Schover et al. [37••]; US	Sexually dysfunctional survivors of localized breast or gynecological cancer; *N* = 72 (N_INT_ = 36)	CBT + counselling (*Tendrils;* 3 face-to-face counseling sessions); CBT self-help; 12 weeks	Web site included text, graphics, animations, and multicultural photographs and clipart; Counselors guided women through the web site and discussed homework.	FSFI (A), MSIQ, BSI-18, QLACS; 3 and 6 months	*Post-treatment effects*: Combined groups: FSFI higher: *p* < 0.001 MSIQ higher: *p* < 0.001 BSI-18 lower: *p* = 0.001 QLACS lower: *p* < 0.001 CBT + counselling: FSFI higher: *p* < 0.001 MSIQ higher: *p* < 0.001 BSI-18: n.s. QLACS: n.s. CBT self-help: FSFI higher: *p* < 0.054 MSIQ higher: n.s. BSI-18 lower: *p* = 0.011 QLACS lower: *p* = 0.008 *Posttreatment to 3- and 6-month follow-up effects*: CBT + counselling: FSFI: n.s. (n.s.) MSIQ lower: n.s. (*p* = 0.018) BSI-18: n.s. (n.s.) QLACS: n.s. (n.s.) CBT self-help: FSFI lower: *p* = 0.050 (n.s.) MSIQ: n.s. (n.s.) BSI-18: n.s. (n.s.) QLACS: n.s. (n.s.)
Hummel et al. [34]^1^; The Netherlands	Survivors of breast cancer with DSM-IV diagnosis of sexual dysfunction; *N* = 160 (N_INT_ = 80)	CBT program (*KIS*); delayed treatment; waitlist; 24 weeks	Five selected from ten preprogrammed online modules; guidance by sexologist in 20 online text chat sessions with the female participant, and preferably also with male partner.	SAQ (A), FSFI (A), FSDS-R (A), PAIR (A), EORTC-QLQ-BR, FACT-ES ESS-18, HADS, SF-36; 3 and 9 months	NA

^1^Protocol paper, no data published yet

*FSFI* Female Sexual Function Index, *DASS* Depression, Anxiety, Stress Scale, *SFS* Sexual Function Scale, *PAIR* Personal Assessment of Intimacy in Relationships, *FSDS-R* Female Sexual Distress Scale–Revised, *SFQ* Sexual Function Questionnaire, *BDI* Beck Depression Inventory, *MSIQ* Menopausal Sexual Interest Questionnaire, *BSI-18* Brief Symptom Inventory-18, *QLACS* Quality of Life in Adult Cancer Survivors Scale, *SAQ* Sexual Activity Questionnaire, *EORTC-QLQ-BR* European Organization for Research and Treatment-QOL questionnaire and breast cancer-specific module (body image scale), *FACT-ES ESS-18* Functional Assessment of Cancer Therapy – Endocrine Subscales, *HADS* Hospital Anxiety and Depression Scales, *SF-36* MOS 36-item short-form health survey, *η* ^*2*^_*part*_ partial η^2^; *Effect* effect of score changes over time in Linear mixed models (LMM) analysis, *NA* not applicable

**Table 2:** Characteristics of internet-based interventions for women’s sexual dysfunctions (Webb et al. 2010, JMIR)

	Jones and McCabe [35]; McCabe and Jones [36]	Hucker and McCabe [31-33]	Schover et al. [37••]	Wiljer et al. [38]	Hummel et al. [34]
Theoretical Basis					
Sexual response cycle model	X	X	X		X
Cognitive theory (in CBT)	X	X			X
Learning theory (in CBT)	X	X			X
Mindfulness theory		X			
Supportive–expressive group therapy model				X	
Use of Theory					
Theory/predictors used to select intervention recipients	X	X	X	X	X
Theory/predictors used to develop intervention techniques	X	X	X		X
Theory/model of behavior mentioned	X	X		X	X
At least one of the intervention techniques is linked to theory	X	X	X		X
Intervention based on single theory			X	X	
Behavior Change Technique					
Sensate focus exercises	X	X	X		X
Communication skills training	X	X	X		X
Hurdle requirement before entry of next module	X				
Challenging negative automatic thoughts		X			X
Psychoeducation		X	X	X	X
Mindfulness exercises		X			
Relapse prevention		X			X
Interviews on video			X		
Role playing examples on video			X		
Homework assignments					X
Discuss intimacy issues with partner					X
Task concentration training					X
Exposure techniques					X
Provide feedback on performance	X	X	X		X
Provide instruction			X		
Provide normative information about others’ behavior			X		
Mode of Delivery: Communicative Functions					
Access to advisor to request advice	X	X	X	X	X
Scheduled contact with therapist	X	X	X		X
Unlimited client-initiated contact	X				X
Peer-to-peer access				X	
Mode of Delivery: Additional Modes					
Email	X				
Text chat group conferencing		X			
Individual text chat					X
Face-to-face counseling sessions			X		

### Theoretical Background and Theory-Based Strategies and Behavior Change Techniques Used in Internet-Based Interventions

The theoretical foundations of three of five interventions [31, 34, 35] in the included studies align with the mainstream of current interventions for common mental disorders: cognitive theory and learning theory [40, 41]. Intervention ingredients in these studies were derived from these conceptual frameworks, including challenging negative automatic thoughts, relapse prevention, role playing exercises, exposure techniques, and provision of feedback on performance in homework (see Table 2). Sexual response cycle theory [8, 42] was used as an important theoretical background in four interventions [31, 34, 35, 37••]; the central position in these interventions of progressive sensate focus exercises traces back to this—specifically sexological—theoretical model. One intervention [31] had a significant background in mindfulness theory, which is often integrated with cognitive-behavioral approaches [43, 44]. One RCT using qualitative methods of evaluation [38] was based on the supportive–expressive group therapy model for cancer survivors [45]. Some therapeutic elements were included in several interventions without an explicit rationale provided. Communication skills training or instruction for improved communication with the partner were included in four interventions, without any specific reference to a theoretical background, such as cybernetics or systems theory. Psychoeducation was mentioned in three interventions, and relapse prevention in two interventions.

### Modes of Delivery of Internet-Based Interventions

In four interventions [31, 34, 35, 37••], participants could use therapeutic elements that were accessible on a secured Web site. The Web-based part of one intervention [37••] contained information on a wide variation of problem-related topics but did not give specific instructions on how to use this information. Three interventions [31, 34, 35] comprised a series of therapeutic modules, containing psychoeducation about specific problem elements. They also provided instructions for the use of techniques to modify cognitions or behaviors that serve to maintain the sexual problem, such as challenging negative automatic thoughts, or reducing performance demand by performing sensate focus exercises. One intervention [38] did not offer “stand-alone” Web site content but relied fully on the exchange of information among participants in asynchronous text chats (discussion board/forum), moderated by sexual health care professionals.

All interventions, except for the Wiljer et al. [38] intervention, included scheduled meetings with a professional to provide guidance and support for the use of the Web-based content. In addition, two interventions [34, 35] allowed participants unlimited text-based contact with the therapist on the participant’s initiative. All interventions allowed some form of access to a professional for requesting advice. Peer-to-peer interaction was enabled in one study [38] using a group text chat facility.

### Effects of Internet-Based Interventions for Women’s Sexual Dysfunctions

The efficacy of three interventions was compared with delayed treatment or waitlist [31, 34, 35]. Of two interventions [31, 35], comparison of posttreatment gains suggested that different aspects of sexual functioning (FSFI: η2_part_ = 0.56; PAIR sexual intimacy: η2_part_ = 0.26) and relational functioning (PAIR: η2_part_ = 0.39; PAIR emotional intimacy: η2_part_ = 0.08; PAIR communication: η2_part_ = 0.08) were improved during the active intervention. In one study [35], no effects of the intervention on anxiety and mood were found, suggesting that this, dedicated, intervention only had effect on the specific, targeted problem areas. Posttreatment gains regarding sexual and relational functioning were generally maintained until follow-up assessment. Of the third intervention [34], no effect data is yet available.

Of the interventions for women with cancer [37••], two different versions were compared, respectively, with and without additional face-to-face counseling sessions. Both versions were found to have beneficial effects on sexual functioning (FSFI: *p* < 0.001), menopausal complaints (MSIQ: *p* < 0.001), general psychological well-being (BSI-18: *p* = 0.001), and quality of life (QLACS: *p* < 0.001). The blended version was found to have stronger effects on sexual functioning (FSFI: *p* < 0.001), compared to the unassisted version, but the latter had stronger effects on general psychological well-being (BSI-18: *p* = 0.01) and quality of life (QLACS: *p* = 0.008). Posttreatment gains were generally maintained through follow-up measurement at both 3 and 6 months, except for sexual functioning in the unassisted version of the intervention, which deteriorated again at 3-month follow-up (FSFI: *p* = 0.05).

## Conclusions

In a search of the literature for this review of Internet-based interventions for women’s sexual dysfunctions, five interventions were identified, reported in a total of eight publications. The included interventions aimed to offer help to women suffering from a broad range of sexual dysfunctions. Both women who were treated after receiving a diagnosis of breast cancer or gynecological cancer and women in the community without comorbid disease were included in the studies investigating the efficacy of treatment. The interventions were all based on common theoretical models of sexual dysfunction and psychological disorders, or on a theory of group counseling [45], and most ingredients of the interventions were theory-informed. Except for one [38], all interventions offered therapeutic content on a secured Web site that could be utilized within a more or less preprogrammed structure. These same interventions also offered prescheduled and/or participant-initiated contact with a sexual health care professional. Comparisons of the active interventions with a control group showed improvements in sexual functioning as well as relational functioning at the point of termination of the treatment period, whereas the beneficial effects on other domains of (psychological and physical) functioning were equivocal. Improvements at posttreatment were generally maintained for several months after termination of the active intervention period. One intervention [37••] included a face-to-face component, three counseling sessions, whereas all others only employed text exchange, either asynchronous through email [35] or using an online peer-to-peer discussion board [38], or synchronous through real-time text chat [31, 34]. The results of the face-to-face intervention that was blended with a Web-based algorithm were directly compared to the use of the algorithm as a self-help intervention. The direct effects of the blended combination had a stronger effect on sexual functioning at post-treatment and were better maintained after 3-month follow-up than the effects of the self-help intervention. The self-help version, however, had larger effects on general well-being and quality of life that were sustained at 3- and 6-month follow-up. These findings are in line with the outcomes of Internet-based interventions for other mental disorders [4•]. Supplementing Web-based algorithms with some face-to-face support, therefore, seems important for obtaining maximal results, although this issue deserves further investigation. The results of the present review seem to warrant further development of Internet-based interventions for women’s sexual dysfunctions and rigorous investigation of the benefits and risks of this approach. Specifically, investigator’s attention should be directed at identifying predictive factors that help protect potential users of this type of interventions against incorrect self-diagnosis and subsequent failures in finding adequate help for their suffering. These shortcomings suggest that the current potential for dissemination of Internet-based interventions for women’s sexual dysfunctions outside of research settings is still limited. However, this has not withheld service providers, at least in Europe, to offer such interventions on a large scale to the public seeking help for sexual problems. Both in the USA and elsewhere ethical, legal and jurisdictional issues still need to be worked out. The creation of professional guidelines based on solid scientific evidence of the efficacy of interventions, the demarcation of target groups for whom online help is likely to be beneficial, the provision of adequate information to the target populations, and the adherence to such guidelines for reimbursement of treatment by health insurance companies might be helpful in maximizing the efficacy of Internet-based health care and its safe use in the field of sexual dysfunctions.

## References

[1] Emmelkamp PM, David D, Beckers T (2014). Advancing psychotherapy and evidence-based psychological interventions. Int J Meth Psychiatr Res.

[2] Lovejoy TI, Demireva PD, Grayson JL (2009). Advancing the practice of online psychotherapy: an application of Rogers’ diffusion of innovations theory. Psychother.

[3] Månsson KNT, Ruiz ES, Gervind E (2013). Development and initial evaluation of an internet-based support system for face-to-face cognitive behavior therapy: a proof of concept study. J Med Internet Res.

[4.•] Palmqvist B, Carlbring P, Andersson G (2007). Internet-delivered treatments with or without therapist input: does the therapist factor have implications for efficacy and cost?. Exp Rev Pharmacoecon Outcomes Res.

[5.•] Andersson G. Internet-delivered psychological treatments. Annu Rev Clin Psychol. 2015; doi:10.1146/annurev-clinpsy-021815-093006. This recent review provides a comprehensive overview of Internet-based interventions for common mental disorders. 10.1146/annurev-clinpsy-021815-093006 26652054

[6] Hall P (2004). Online psychosexual therapy: a summary of pilot study findings. Sex Rel Ther.

[7] van Lankveld J, Mevissen FEF, Wylie KR (2015). Bibliotherapy and internet-based programs for sexual problems. ABC of sexual health.

[8] Masters WH, Johnson VE (1970). Human sexual inadequacy.

[9] van Lankveld J, Watkins PL, Clum GA (2008). Self-help therapies for sexual dysfunctions. Handbook of self-help therapies.

[10] Andersson G, Titov N (2014). Advantages and limitations of internet-based interventions for common mental disorders. World Psychiatr.

[11] Andersson G, Cuijpers P, Carlbring P (2014). Guided internet-based vs. face-to-face cognitive behavior therapy for psychiatric and somatic disorders: a systematic review and meta-analysis. World Psychiatr.

[12] Arnberg FK, Linton SJ, Hultcrantz M (2014). Internet-delivered psychological treatments for mood and anxiety disorders: a systematic review of their efficacy, safety, and cost-effectiveness. PLoS One.

[13.••] Webb TL, Joseph J, Yardley L (2010). Using the internet to promote health behavior change: a systematic review and meta-analysis of the impact of theoretical basis, use of behavior change techniques, and mode of delivery on efficacy. J Med Internet Res.

[14] Hedman E, Ljotsson B, Lindefors N (2012). Cognitive behavior therapy via the Internet: a systematic review of applications, clinical efficacy and cost-effectiveness. Exp Rev Pharmacoecon Outcomes Res.

[15] Nordgren LB, Hedman E, Etienne J (2014). Effectiveness and cost-effectiveness of individually tailored internet-delivered cognitive behavior therapy for anxiety disorders in a primary care population: a randomized controlled trial. Behav Res Ther.

[16] Warmerdam L, Smit F, van Straten A (2010). Cost-utility and cost-effectiveness of internet-based treatment for adults with depressive symptoms: randomized trial. J Med Internet Res.

[17] Hedman E, Andersson E, Lindefors N (2013). Cost-effectiveness and long-term effectiveness of internet-based cognitive behaviour therapy for severe health anxiety. Psychol Med.

[18] Gerhards SA, de Graaf LE, Jacobs LE (2010). Economic evaluation of online computerised cognitive-behavioural therapy without support for depression in primary care: randomised trial. Br J Psychiatr.

[19] Borrelli B, Ritterband LM (2015). Special issue on ehealth and mhealth: Challenges and future directions for assessment, treatment, and dissemination. Health Psychol.

[20] Andersson G, Carlbring P, Berger T (2009). What makes internet therapy work?. Cogn Behav Ther.

[21.•] Boettcher J, Berger T, Renneberg B (2012). Does a pre-treatment diagnostic interview affect the outcome of internet-based self-help for social anxiety disorder? a randomized controlled trial. Behav Cogn Psychother.

[22] Ellis A (1962). Reason and emotion in psychotherapy.

[23] Janssen E, Everaerd W, Spiering M (2000). Automatic processes and the appraisal of sexual stimuli: toward an information processing model of sexual arousal. J Sex Res.

[24] Nobre PJ (2010). Psychological determinants of erectile dysfunction: testing a cognitive-emotional model. J Sex Med.

[25] Barlow DH (1986). Causes of sexual dysfunction: the role of anxiety and cognitive interference. J Consult Clin Psychol.

[26] Barbach LG (1974). For yourself: the fulfillment of female sexuality.

[27] van Lankveld J (1998). Bibliotherapy in the treatment of sexual dysfunctions: a meta-analysis. J Consult Clin Psychol.

[28] van Lankveld J (2009). Self-help therapies for sexual dysfunction. Ann Rev Sex Res.

[29] Hektner JM, Schmidt JA, Csikszentmihalyi M (2007). Experience sampling method: measuring the quality of everyday life.

[30] Mehl MR, Conner TS (2012). Handbook of research methods for studying daily life.

[31] Hucker A, McCabe MP (2014). An online, mindfulness-based, cognitive-behavioral therapy for female sexual difficulties: impact on relationship functioning. J Sex Mar Ther.

[32] Hucker A, McCabe MP (2014). A qualitative evaluation of online chat groups for women completing a psychological intervention for female sexual dysfunction. J Sex Mar Ther.

[33] Hucker A, McCabe MP (2015). Incorporating mindfulness and chat groups into an online cognitive behavioral therapy for mixed female sexual problems. J Sex Res.

[34] Hummel SB, van Lankveld JJ, Oldenburg HS (2015). Internet-based cognitive behavioral therapy for sexual dysfunctions in women treated for breast cancer: design of a multicenter, randomized controlled trial. BMC Cancer.

[35] Jones LM, McCabe MP (2011). The effectiveness of an internet-based psychological treatment program for female sexual dysfunction. J Sex Med.

[36] McCabe MP, Jones LM (2013). Attrition from an internet-based treatment program for female sexual dysfunction: who is best treated with this approach?. Psychol Health Med.

[37.••] Schover LR, Yuan Y, Fellman BM (2013). Efficacy trial of an internet-based intervention for cancer-related female sexual dysfunction. J Nat Compreh Cancer Netw.

[38] Wiljer D, Urowitz S, Barbera L (2011). A qualitative study of an internet-based support group for women with sexual distress due to gynecologic cancer. J Cancer Educ.

[39] Wiegel M, Meston C, Rosen R (2005). The Female Sexual Function Index (FSFI): cross-validation and development of clinical cutoff scores. J Sex Mar Ther.

[40] Mineka S, Zinbarg R (2006). A contemporary learning theory perspective on the etiology of anxiety disorders: it’s not what you thought it was. Am Psychol.

[41] Clark DA, Beck AT (2010). Cognitive theory and therapy of anxiety and depression: convergence with neurobiological findings. Tr Cogn Sci.

[42] Masters WH, Johnson VE (1966). Human sexual response.

[43] Brotto LA, Goldmeier D (2015). Mindfulness interventions for treating sexual dysfunctions: the gentle science of finding focus in a multitask world. J Sex Med.

[44] Piet J, Hougaard E (2011). The effect of mindfulness-based cognitive therapy for prevention of relapse in recurrent major depressive disorder: a systematic review and meta-analysis. Clin Psychol Rev.

[45] Spiegel D, Classen C (2000). Group therapy for cancer patients: a research-based handbook of psychosocial care.

